# Self-medication in university students from the city of Rio Grande, Brazil

**DOI:** 10.1186/1471-2458-12-339

**Published:** 2012-05-08

**Authors:** Marília Garcez Corrêa da Silva, Maria Cristina Flores Soares, Ana Luiza Muccillo-Baisch

**Affiliations:** 1 Department of Health Sciences, Universidade Federal do Rio Grande (FURG), Rua General Osório, s/n Caixa Postal 474, CEP 96201-900 Centro, Rio Grande, RS, Brazil

## Abstract

**Background:**

Self-medication is the use of medication without prescription, orientation, or supervision of a physician or dentist. Self-medication might become a serious health problem. The purpose of this study was to identify the prevalence and factors associated with self-medication among first and last-year students enrolled in healthcare and non-healthcare programs.

**Methods:**

A cross-sectional study was conducted at Universidade Federal do Rio Grande (FURG), state of Rio Grande do Sul, Brazil. Of 830 students in the sample, 95% answered the questionnaire – 789 students enrolled in 10 undergraduate programs. Mean age was 22 ± 6.17 years. The students answered a questionnaire covering socio-economic and demographic variables, use of medication, and medication knowledge. Information was collected on the conditions treated with medication, the medications used, and attitude towards self-medication.

**Results:**

Of 789 students, 86.4% self-medicated (88.5% of 446 healthcare students). There were no significant differences in self-medication between healthcare and non-healthcare students, nor between first and last-year students. Bivariate and multivariate analyses showed a significant association between self-medication and having children (*p* = 0.01), having a home pharmacy (*p* < 0.001) and adequate medication knowledge (*p* = 0.01). The most frequently used active ingredients were acetaminophen (paracetamol), dipyrone, aspirin, phytotherapic compounds, and tea. Illicit drug use was significantly associated with self-medication in the multivariate analysis.

**Conclusion:**

The fact that being a healthcare student was associated with higher medication knowledge, but not with less self-medication, suggests that medication knowledge might contribute to increase self-medication. This should be taken into account when designing educational interventions relating to self-medication.

## Background

The patterns of medication use are an important health indicator. Knowledge concerning these patterns helps identify and determine the prevalence of diseases affecting specific populations, and also provides information about how therapeutic resources are used [1]. In this context, self-medication is an important issue, which may delay diagnosis and facilitate the emergence of resistant microorganisms and iatrogenic illnesses [2]. Even if the drugs are used correctly, self-use may be associated with side effects and increased chance of drug interactions, including drug-alcohol interactions. It may also affect adherence to treatment and quality of life [3].

Self-medication is defined by the Brazilian Health Surveillance Agency (ANVISA) as the use of drugs without prescription, guidance, or follow-up by a physician or dentist [4]. In Brazil, self-medication is common in the general population [5], and at least 35% of the volume of drugs purchased are for self-medication [6]. Drug sales in Brazil are regulated by ANVISA. Drugs can only be purchased in pharmacies, but the prescription is retained at the pharmacy only for certain types of drugs (e.g. psychotropic medication). All other sales are based on over-the-counter (OTC) drugs (aspirin, acetaminophen), which do not require a prescription, or drugs that require only presenting a prescription (anti-inflammatory drugs, antihypertensive drugs), that is, the buyer can simply show the prescription, but it is not retained at the pharmacy.

Therefore, the present study was conducted to determine the prevalence of self-medication among first and last-year students enrolled in healthcare and non-healthcare programs in a Brazilian university.

## Methods

Between April 2010 and July 2010, a descriptive, cross-sectional study was conducted with students from the Federal University of Rio Grande (FURG). Data concerning demographic characteristics, medication use habits, and self-medication were collected through a self-reported questionnaire with open and closed-ended questions. Prior to answering the questionnaire, the students were given a brief explanation about the intention of the study. Also, the ANVISA definition of self-medication [4] was read out loud. The choice of questions, as well as the list of symptoms that might have been self-medicated, was based on the findings of previous studies [7-12]. The last part of the questionnaire, covering medication knowledge, was adapted to the Brazilian context from the method described by Isacson & Bingefors [13], which has been previously used to assess self-medication [11]. Medication knowledge was evaluated by the number of correct answers to six questions and classified as follows: adequate (5–6 correct answers), average (2–4 correct answers), or poor (0–1 correct answer). A pilot study was conducted in March 2010 with 30 students from all fields to test the instrument, determine application time, and clarify possible questions from the students.

The following healthcare programs were evaluated: Biological Sciences, Physical Education, Nursing, Medicine, and Psychology. Non-healthcare programs-Visual Arts, Food Engineering, Geography, Law, and Marine Biology-were similar to healthcare programs in terms of class schedules (day or evening courses) and age of students. The sample included all first and last-year students enrolled in these programs.

Questionnaires were given at the start of the semester in the beginning of class sessions. If a student was not in class on the day of the interview, the investigators returned to each session until the day of final exams. Of 830 students in the sample, 95% answered the questionnaire (446 healthcare students and 343 non-healthcare students).

All medications cited were classified according to active ingredient (Anatomical Therapeutic Chemical (ATC) classification system [14]). Any brand names cited were coded for analysis according to active ingredient. The study outcome was self-reported self-medication.

A hierarchical model was used to determine self-medication risk factors. The model allowed the contribution of each hierarchical level to be quantified and prevented underestimation of risk effects. It also yielded a simplified explanatory model, which contributed to the understanding of the outcome. The following variables were included in the theoretical model based on a review of the literature: first level-socioeconomic and demographic status (age, sex, skin color, income, employment, having or not a partner, housing arrangement, having or not children, parents’ age and level of education); second level-life style (smoking, use of illicit drugs, other health-protective habits), health perception (self-perception of current health status), use of healthcare services (last medical appointment, presence of home pharmacy-drugs stored at home at a designated place referred to herein as “home pharmacy”); third level-field of study (healthcare or non-healthcare); forth level-class (first or last-year); fifth level-medication knowledge.

A bivariate analysis was carried out to evaluate the crude effect of each independent variable on self-medication (study outcome). A multivariate analysis was then carried out to evaluate the effect of adjusted variables on each other within each block, according to the theoretical model and hierarchical levels. In order to prevent the exclusion of possible confounding factors, the variables with p ≤ 0.2 in any of the levels were kept in the model until the end, even if they lost significance with the introduction of new variables. A p ≤ 0.05 was defined as significant for all the analyses.

Data were doubled-entered in EPI Info (version 6.04, Centers for Disease Control and Prevention, Atlanta, GA). Statistical analysis was carried out with STATA 10 (StataCorp, College Station, TX). The strength of the association between the variables was estimated by prevalence ratios and 95% confidence intervals (95% CI). Chi-square tests were used to determine the statistical significance of associations in bivariate analyses. Poisson regression was to estimate crude and adjusted prevalence ratios. Because some questions allowed multiple answers, the sum of the percentages is not always 100%.

The study protocol was approved by the Research Ethics Committee at Universidade Federal do Rio Grande (CEPAS-FURG). Participation was strictly voluntary, and all participants had the right to withdraw from the study at any time. Confidentiality and anonymity were ensured. The questionnaire was applied after signature of an informed consent form, as required by Brazilian regulations [15].

## Results

A total of 789 healthcare and non-healthcare students answered the questionnaires and were included in the analysis. Of these, 485 (62.5%) were female, 606 (77%) were not employed, 663 (84%) did not have a partner, and only 172 (22%) had their own income. The sociodemographic characteristics of participants are described in Table 1.

**Table 1:** Sociodemographic profile of first- and last-year healthcare and non-healthcare students (n = 789)

**Variable**	**Healthcare**		**Non-healthcare**		**Total**
	**First-year**	**Last-year**	**First-year**	**Last-year**	
Age, n (%)					
> 30 years	19 (7.1)	14 (7.9)	28 (12.8)	20 (16.1)	81 (10.3)
20–30 years	159 (59.3)	157 (88.2)	93 (42.5)	103 (83.1)	512 (64.9)
≤ 19 years	90 (33.6)	7 (3.9)	98 (44.7)	1 (.8)	196 (24.8)
Total	268	178	219	124	789
Sex, n (%)					
Female	188 (70.4)	112 (63.3)	133 (61.0)	58 (46.8)	491 (62.5)
Male	79 (29.6)	65 (36.7)	85 (39.0)	66 (53.2)	295 (37.5)
Total	267	177	218	124	786
Race, n (%)					
White	230 (86.8)	156 (88.6)	178 (82.4)	100 (80.6)	664 (85.0)
Non-white	35 (13.2)	20 (11.4)	38 (17.6)	24 (19.4)	117 (15.0)
Total	265	176	216	124	781
Housing arrangement, n (%)					
Family	170 (63.4)	96 (54.2)	128 (58.4)	74 (60.2)	468 (59.5)
Friends	53 (19.8)	33 (18.6)	43 (19.6)	30 (24.4)	159 (20.2)
Alone	34 (12.7)	37 (20.9)	31 (14.2)	15 (12.2)	117 (14.9)
n/a	11 (4.1)	11 (6.2)	17 (7.8)	4 (3.3)	43 (5.5)
Total	268	177	219	123	787
Children, n (%)					
No	241 (89.9)	165 (92.7)	185 (84.5)	107 (86.3)	698 (88.5)
Yes	18 (6.7)	12 (6.7)	28 (12.8)	14 (11.3)	72 (9.1)
Total	268	178	219	124	789
Paternal age, n (%)					
37–50 years	108 (45.2)	37 (23.3)	86 (45.5)	38 (37.3)	269 (39.0)
51–89 years	131 (54.8)	122 (76.7)	103 (54.5)	64 (62.7)	420 (61.0)
Total	239	159	189	102	689
Maternal age, n (%)					
34–50 years	162 (63.3)	70 (40.7)	129 (62.3)	52 (44.1)	413 (54.8)
51–88 years	94 (36.7)	102 (59.3)	78 (37.7)	66 (55.9)	340 (45.2)
Total	256	172	207	118	753
Paternal education, n (%)					
< 8 years	57 (21.3)	27 (15.2)	50 (23.0)	21 (16.9)	155 (19.7)
8–11 years	118 (44.2)	80 (44.9)	89 (41.0)	61 (49.2)	348 (44.3)
> 11 years	92 (34.5)	71 (39.9)	78 (35.9)	42 (33.9)	283 (36.0)
Total	267	178	217	124	786
Maternal education, n (%)					
< 8 years	57 (21.3)	27 (15.2)	50 (23.0)	21 (16.9)	155 (19.7)
8–11 years	92 (34.5)	71 (39.9)	78 (35.9)	42 (33.9)	283 (36.0)
> 11 years	118 (44.2)	80 (44.9)	89 (41.0)	61 (49.2)	348 (44.3)
Total	267	178	217	124	786

Of this sample, 86.4% reported self-medicating, 58% were healthcare students, and 61% were first-year students. Mean age was 22 ± 6.17 years.

As shown in Table 2, illicit drug use was referred by 8.8% (67) of the students; 64 (95.5%) used marijuana, 16 (23.8%) used LSD, 14 (20.8%) used cocaine, 6 (8.9%) used ecstasy, and 6 (8.9%) used other drugs (amphetamines, inhaled ethyl chloride, and hashish). Table 2 also shows the distribution of students according to life style, health perception, and use of healthcare services. Six hundred and sixty-eight students (87%) had a home pharmacy.

**Table 2:** Life style, health perception and healthcare-related matters

	**n**	**%**
Life style and health perception		
Smoker		
No	693	90
Yes	74	10
Drug user		
No	694	91
Yes	67	9
Health-protective measures		
Healthy eating or exercise	447	56.9
Healthy eating and exercise	209	26.6
None	129	16.4
Health perception		
Good	443	56.2
Excellent	239	30.3
Could be better	106	13.5
Use of healthcare services		
Last medical appointment		
30–60 days	363	46
6–12 months	258	33
More than 1 year ago	65	8
Doesn’t remember	102	13

Medication knowledge was adequate in 122 participants (15.5%), average in 463 (58.8%), and poor in 202 (25.7%). The difference between healthcare and non-healthcare students concerning medication knowledge was statistically significant: 89 healthcare (vs. 33 non-healthcare) students had adequate knowledge; 271 healthcare (vs. 192 non-healthcare) students had average knowledge; and 85 healthcare (vs. 117 non-healthcare) students had poor medication knowledge (*p* < 0.001).

The overall reasons for self-medication were headache (89.7%), cold (82.9%), sore throat (58.1%), fever (56.2%), menstrual cramps (47.6%), muscle pain (41.0%), cough (36.4%) and heartburn (29.4%); and also stomachache (27.1%), nausea (26.4%), vomit (22.3%), allergy (21.2%) and intestinal colic (14%). Self-medication was statistically higher among healthcare students in most cases (Table 3).

**Table 3:** Reasons for self-medication among healthcare and non-healthcare students

	**Healthcare**		**Non-healthcare**		
**Variable**	**n**	**%**	**n**	**%**	***P***
Fever	244	62.10	137	48.10	<0.05
Menstrual cramps	206	52.40	117	41.10	0.00
Muscle pain	176	44.80	102	35.80	0.02
Nausea	127	32.00	52	18.20	<0.05
Stomachache	119	30.30	65	22.80	0.03
Vomit	98	24.90	53	18.60	0.05
Allergy	96	24.40	48	16.80	0.02
Intestinal pain	71	18.10	24	8.40	<0.05
Cough	142	36.10	105	36.80	0.04

The following socioeconomic variables were significantly associated with self-medication in the bivariate analysis (Table 4): employment (*p* = 0.02), having a partner (*p* = 0.03), having children (*p* = 0.00), age (*p* = 0.02) and male sex (*p* = 0.01). In the healthcare service block, existence of a home pharmacy (*p* = 0.00) was significantly associated with self-medication. No variable in the life style and health perception block was significantly associated with self-medication. In the bivariate analysis, being or not a healthcare student was close to be significantly (*p* = 0.06) associated with self-medication. The same was true for last-year students from healthcare vs. non-healthcare programs (*p* = 0.01). Finally, poor medication knowledge was significantly associated with less self-medication (*p* = 0.00).

**Table 4:** Adjusted and crude analysis of the variables associated with self-medication

	**Bivariate Analysis**			**Multivariate Analysis**		
**Variable**	**PR**	**(95%CI)**	***p***	**PR**	**(95% CI)**	***p***
Employment			0.02		0.41	
No	1.00			1.00		
Yes	0.91	(0.842–0.988)		0.96	(0.880–1.055)	
Partner			0.03			0.75
No	1.00			1.00		
Yes	0.90	(0.821–0.992)		1.02	(0.920–1.22)	
Children			0.00			0.014
No	1.00			1.00		
Yes	0.80	(0.690–0.933)		0.83	(0.712–0.963)	
Age			0.02			0.45
> 30	1.00			1.00		
20–30	1.22	(1.05–1.41)				
≤ 19	1.24	(1.06–1.44)		1.03	(0.962–1.092)	
Sex			0.01			0.03
Female	1.00			1.00		
Male	0.92	(0.87–0.98)		0.93	(0.879–0.993)	
Illicit drug use			0.38			0.05
No	1.00			1.00		
Yes	1.00	(0.95–1.13)		1.09	(1.001–1.180)	
Home pharmacy			0.00			0.00
No	1.00			1.00		
Yes	1.40	(1.21–1.62)		1.39	(1.201–1.606)	

First and second level in the hierarchical model.

95%CI = 95% confidence interval; PR = prevalence ratio.

Multivariate analysis revealed that in the first level of analysis, sex (*p* = 0.03) and having children (*p* = 0.01) were statistically associated with self-medication, as well as illicit drug use (*p* = 0.05) and having a home pharmacy (*p* = 0.00). In the third level, the association between self-medication and being a non-healthcare student was close to significance (*p* = 0.07) and the variable was kept in the model. Poor medication knowledge remained statistically associated with less self-medication in the multivariate analysis (*p* = 0.00).

A total of 2,348 active ingredients were cited, an average of 3.45 medications per student. The most common were acetaminophen formulations (478, 20.3%), dipyrone formulations (437, 18.6%), aspirin (146, 6.2%), phytotherapeutic formulations, and herbal teas.

When asked about attitude towards self-medication, 605 (81.9%) students replied that they discouraged friends and relatives from self-medication. The attitude towards self-medication was significantly different between healthcare and non-healthcare students (85.8 vs. 76.6%, respectively; *p* < 0.001), i.e., a larger number of healthcare students discouraged friends and relatives from self-medication. Advice concerning self-medication was obtained from the sources described in Table 5. The explanations for self-medication are listed in Table 6.

**Table 5:** Sources of advice on self-medication

**Source**	**n**	**%**
Family	408	53.1
Pharmacist/clerk	398	51.7
Old prescription	311	40.4
Own decision	227	29.5
Media (magazines and Internet)	156	20.3
Friends/neighbors	152	19.7
Class	148	19.2
Books	92	12.0
Others	59	6.8

**Table 6:** Explanations for self-medication

**Explanation**	**n**	**%**
I have already had the symptom and I know what to “take”	386	57.2
There is no need to see a doctor because of a simple disease	299	44.3
Quick relief	234	34.7
The physician will prescribe me the same medication	206	30.5
Economy of time	180	26.7
Economy of money	124	18.4
Unavailability of health service	41	6.1
I do not trust in health service	13	1.9
Opportunity of learning	8	1.2

## Discussion

In the present study, 86.4% of the total students reported self-medicating. This result is similar to that reported in studies conducted with university students in the Palestine (98%) [11] and Slovenia (92.3%) [16]. It is interesting to note that a Brazilian study conducted with university healthcare and non-healthcare students in the city of Recife [8] showed that 57.7% declared not to self-medicate. As shown in a previous study, healthcare-related education in students led to more responsible self-medication [16].

Previous studies comparing university healthcare to non-healthcare programs include a small number of healthcare students [10,11,17]. In the present study, in which the multivariate analysis revealed a relationship between self-medication and several protection and predisposing factors, a larger number of healthcare students (56.5%) was included, as was also the case with the study by Klemenc-Ketis et al. [16].

Several aspects influence self-medication, such as education, family, advertising, legislation, having previous experience with a symptom or disease, importance attributed to a disease, and economic issues [9]. Also, self-medication, as well as seeking advice from friends and relatives, might be a way of overcoming the obstacles to medical care, or else result from dissatisfaction with medical care [18]. The World Health Organization (WHO) supports self-medication as a means to reduce costs for the healthcare system and the individual citizens. However, the WHO stresses that self-medication can only be used in countries that are able to provide adequate healthcare and education, and thus empower citizens to self-medicate responsibly [19].

As previously reported [10,16,20], we did not observe any significant differences in prevalence of self-medication by healthcare and non-healthcare students. This contrasts with the results obtained by Sawalha [11] and Sapkota et al. [21], who showed low prevalence of self-medication among healthcare students.

Being a first or last-year student did not affect the outcome. We hypothesized that after a few years in university, students would be more aware of the risks of self-medication, as reported by Sapkota et al. [21]. In that study, being a last-year student was a protection factor for self-medication.

According to Gama et al*.*[22], the structure of questionnaires may affect prevalence estimates. Those authors found that longer questionnaires, including more questions, with specific indications and pharmacological groups resulted in higher prevalence of self-reported self-medication, whereas a shorter questionnaire with open questions resulted in a lower prevalence of self-reported self-medication in the same population. The fact that we employed a long questionnaire, with 62 questions, could explain the higher prevalence of self-reported self-medication we observed. Concerning the demographic and socioeconomic profile, the present sample is similar to those of other studies on self-medication among university students [8,10,11]. Previous results regarding the influence of factors such as sex, age, and socioeconomic status on self-medication are controversial [7,12].

In the present study, having children, being male, being employed and having a partner, were significantly associated with self-medication, but the two former factors lost significance after adjustment. Having children and being male were identified as protection factors against self-medication.

In Croatia, Alijinović-Vucić et al. [23] reported the existence of a home pharmacy in 68.3% of the households surveyed in a study about self-medication in medical students. The fact that 87% of our students also mentioned having a home pharmacy suggests that this factor is a risk factor for self-medication. The home pharmacy was significantly associated with self-medication in the bivariate and multivariate analyses. It might also be associated with the reasons cited for self-medication, of which the first one was “I have already experienced the symptom and know what to take” (57.2%). It may reflect a usual behavior and the repeated use of an old prescription. The storage of medication at home with free access and easy visualization of the products is a risk factor for self-medication [24]. Receiving advice about self-medication mainly from the family (53.1%) and the reuse of an old prescription (40.4%) contribute to the risk posed by home pharmacies. This suggests easy access to medication and a culturally inherited acceptance of self-medication, as pointed out by Abahussain et al. [7]. In the present study, additional explanations for self-medication were cited by the students, including “There is no need to see a doctor because of a simple disease” and “Quick relief,” among others. These explanations could also be supported by the existence of a home pharmacy.

As to life style, illicit drug use was found to be a risk factor for self-medication. An association between self-medication and illicit drug use has not been previously reported. Marijuana was the drug most frequently used by students (96%), and was associated with use of other illicit drugs in 32.8% of the cases.

Healthcare students had significantly more knowledge about medication than non-healthcare students. Similar results were obtained by Sawalha [11]. Medication knowledge was significantly associated with the outcome in both the bivariate and multivariate analyses. The lack of adequate medication knowledge seems to have made the students more cautious, leading to less self-medication. In a study conducted with medical students from Bahrein, those who had more knowledge about medication reported self-medicating more [9].

As to the type of medication used, as mentioned in the literature [7,8,10-12], acetaminophen was the most common active ingredient. This could be justified by the reasons cited for self-medication, including headache, colds, and sore throat, as reported by other investigators [8,9]. Interestingly, for specific conditions such as menstrual cramps, nausea, and vomit, the percentage of healthcare students self-medicating was significantly higher, as also observed by Sawalha [11] using multiple logistic regression.

Most students who answered the questionnaire (81.9%) declared having discouraged friends and relatives from self-medicating. In the comparison between areas, we observed a significantly higher percentage of reported discouragement among healthcare students. This result contradicts the high prevalence of self-medication in this sample. Another study conducted with medical students shows that self-medication was used by most the students; those authors suggest that healthcare students feel more confident self-prescribing [9].

The present study has limitations that need to be addressed. First of all, because the sample refers to a specific university and a specific geographic area, it cannot be generalized. Also, chronic diseases, which are more often associated with self-medication, were not assessed. Nevertheless, we believe that the present results will be useful for other investigators as well as healthcare programs designing interventions relating to self-medication. Knowledge of the advantages, disadvantages and consequences of self-medication is important to raise awareness about the seriousness of prescribing. The behavior of students may influence his or her attitude towards the patient in professional practice.

## Conclusions

The questionnaire we employed to assess self-medication was useful to characterize the present sample, the pattern of medication use, and the level of medication knowledge. Even though the prevalence of self-medication was high in this student sample, it was within the range observed in previous studies. There was no significant difference between healthcare and non-healthcare students regarding self-medication.

Factors such as being male, having children, and having average or poor medication knowledge significantly influenced self-medication, as protection factors. Illicit drug use and the existence of a home pharmacy were risk factors for self-medication. Acetaminophen was the most usually employed medication, especially to treat headaches, colds, sore throat, and fever.

Most students, and especially healthcare students, discouraged their friends and relatives from self-medicating. Cultural inheritance is considered to be an important way of transmitting knowledge; it is therefore necessary to incorporate cultural practices that encourage the safe use of medication.

In summary, the fact that being a healthcare student was associated with higher medication knowledge, but not with less self-medication, suggests that medication knowledge might contribute to increase self-medication. This should be taken into account when designing educational interventions relating to self-medication.

## Competing interests

The authors declare that they have no competing interests.

## Authors’ contributions

MCG Designed and conducted the study, collected and analyzed the data, and drafted the article. MCFS participated in the design of the study, performed the statistical analysis and revised the manuscript critically. ALMB participated in the design of the study and revised the manuscript critically. All authors read and approved the final manuscript.

## Pre-publication history

The pre-publication history for this paper can be accessed here:

http://www.biomedcentral.com/1471-2458/12/339/prepub
